# Microwave Dielectric Permittivity of Nanostructured RMn_2_O_5_ Manganate, R_2_Ti_2_O_7_ Titanate, and LiCoPO_4_ and LiNi_0.5_Co_0.5_PO_4_ Orthophosphate Composites

**DOI:** 10.3390/nano15130995

**Published:** 2025-06-26

**Authors:** Anatoly B. Rinkevich, Dmitry V. Perov, Evgeny A. Kuznetsov, Maria S. Stenina

**Affiliations:** M.N. Miheev Institute of Metal Physics UB RAS, Sofia Kovalevskaya St., 18, 620108 Ekaterinburg, Russia; peroff@imp.uran.ru (D.V.P.); kuzeag@mail.ru (E.A.K.); ms-stenina@yandex.ru (M.S.S.)

**Keywords:** nanocomposites, artificial opal, manganates, titanates, orthophosphates, microwaves, transmission and reflection coefficients, complex permittivity, dielectric relaxation

## Abstract

The complex dielectric permittivity has been studied with the waves of millimeter wavelength for rare earth manganate and titanate and LiCoPO_4_ and LiNi_0.5_Co_0.5_PO_4_ orthophosphate composites. The measurements are carried out at frequencies of 26 to 38 GHz via measurements of transmission and reflection coefficients through a plate. A special method on how to extract the real and imaginary parts of dielectric permittivity is applied. Discussion is conducted on a nonmonotonic type of the frequency dependences for both real and imaginary parts of permittivity, and it has been shown that relaxation is non-Debye. The Cole–Cole, Havriliak–Negami, and Kohlrausch–Williams–Watts models cannot also explain the nonmonotonic frequency dependence of the real part of dielectric permittivity. Investigation of the structure and phase composition of nanocomposites has been carried out.

## 1. Introduction

The frequency dependences of the dielectric permittivity of nanostructures and nanocomposites have specific features conditioned by the space limitation of the components of heterogeneous media [1]. As noted in [2], a multi-channel propagation and scattering of phonons and photons is possible in nano-objects that influence the frequency dependences of permittivity. In recent years, an essential interest has attracted investigation of the magnetoelectric effect in multiferroics. This effect is studied specifically in rare earth manganates with the general formula RMn_2_O_5_, where R is a trivalent rare earth ion [3,4,5], and in the orthophosphates LiNiPO_4_ and LiCoPO_4_ [6]. In these systems, a complicated magnetic ordering of the antiferromagnetic type is realized at low temperatures, which can transform into an incommensurate noncollinear type of magnetic structure as temperature increases. The crystal and magnetic structures of several manganates have been investigated in [7] using magnetometry and neutron scattering. The crystal structures of RMn2O5 are orthorhombic at room temperature with the Pbam space group. The magnetic and dielectric properties of five rare earth manganates have been studied in [8]. The magnetic and dielectric phase transitions were demonstrated to occur simultaneously. As a rule, a ferroelectric polarization exists if the spin configuration for Mn^4+^ and Mn^3+^ ions is in the commensurate magnetic phase. The electric polarization in erbium and terbium manganates is studied in [9], and a special attention is drawn to the fact that the electric polarization presents, induced by local polar phase separation domains. In relaxors, standing ferroelectric phonons develop in nanoregions at the frequency when a wavelength equals the polar nanoregion size [10]. High values of the real part of dielectric permittivity have been found for manganates with *R* = Tb, Dy, and Er in the paraelectric phase at temperatures above 78 K at frequencies of 100 Hz to 1 MHz [11]. A correlation between the high dielectric permittivity and the size of *R* ions has been proposed. The influence of doping by yttrium on the complex dielectric permittivity of gadolinium manganate is studied in [12].

The magnetic structure of orthophosphates is investigated in a magnetic field up to 56 T by neutron diffraction and magnetometry [6]. The conditions that allow the magnetoelectric phase in LiNiPO_4_ to exist have been established. These orthophosphate compounds have an orthorhombic crystalline structure (space group *Pnma*) and commensurate antiferromagnetic ground states. A detailed study of the LiNiPO_4_ structure by scanning electron transmission microscopy and X-ray diffraction pointed that a considerable proportion of antisite defects presents in this compound, which may have a pronounced effect on the electrochemical and magnetoelectric properties [13]. The dielectric permittivity of LiNiPO_4_ is examined in [14]. Investigation of dielectric permittivity, performed over the frequency range 10 Hz to 1 MHz at temperatures of 150 to 300 °C, convinced that the relaxation is of non-Debye type. The dielectric permittivity and alternating current conductivity of the LiNiPO_4_ compound were investigated by impedance spectroscopy [15]. The presence of two thermally activated peaks is established from the temperature dependence of impedance. Raman and far-infrared spectra of LiNiPO_4_ were measured and discussed in [16]. It has been established that the value of losses is linked with the distortion of the [NiO_6_] octahedra. A low dielectric constant *ε*′ = 5.18 at a frequency *f* = 17.2 GHz is found in ceramics sintered at 825 °C. The LiNiPO_4_ and LiNi_0.5_Co_0.5_PO_4_ compounds are synthesized by the solid-state reaction method [17]. It is shown that the electrical conductivity of LiNi_0.5_Co_0.5_PO_4_ is higher than that of pure LiNiPO_4_. Zinc-doped LiCoPO_4_ was prepared by the solid-state reaction method [18]. It has been obtained from the temperature dependence of conductivity that both pure and Zn^2+^-doped samples obey the Arrhenius law.

The scenario of dielectric relaxation and the specific features of relaxors are discussed in the review [19]. The high-temperature dielectric properties of BiMn_2_O_5_ manganate are examined in [20], where it has been shown that BiMn_2_O_5_ ceramic demonstrates relaxor behavior. Using the impedance spectroscopy method, it was found that Dy-substituted YMn_2_O_5_ manganate exhibits a non-Debye type of relaxation [21]. The relaxation of dynamically correlated clusters is discussed theoretically in [22]. The Havriliak–Negami and Kohlrausch–Williams–Watts approaches are regarded as more suitable for the micro/mesoscopic relaxing systems. The non-Debye dielectric relaxation is inherent in complex heterogeneous media [23].

As a rule, the high real parts of the dielectric permittivity of rare earth manganates and orthophosphates up to tens of thousands are realized at low frequencies [9,14,21]. A drastic decrease in the real part occurs in GdMn_2_O_5_ and YMn_2_O_5_ at 2–3 kHz [12,21]. In ErMn_2_O_5_, TbMn_2_O_5_ DyMn_2_O_5_ manganates, it sharply decreases at frequencies of 10–100 kHz [11]. In relaxor bismuth manganite, a gradual monotonic decrease in the real part occurs starting from kilohertz to megahertz [20].

The dielectric permittivity of BaZrO_3_-BaTiO_3_ solid solutions is studied in a wide frequency range [24]. The high values of the real part of dielectric permittivity are about 200, remaining up to ~10^11^ Hz. A peak of losses at 100–200 GHz corresponds to a Debye-type relaxation. Additionally, another peak of losses presents in the GHz range. Its frequency dependence satisfies to the Cole–Cole model, and the relaxation time is related to thermally activated processes. In Ba_2_LaTi_2_Nb_3_O_15_ and Ba_2_La_0.5_Nd_0.5_Ti_2_Nb_3_O_15_ niobates, the relaxation takes place mostly at frequencies of MHz and GHz ranges and continues up to ~500 GHz [25]. The real part of permittivity at frequencies of less than 1 kHz is about 800, and it decreases monotonically if the frequency rises. The millimeter-wave dielectric permittivity of nanocomposite rare earth titanates is studied in [26]. The tendency is established that the real part of the dielectric constant of the rare earth nanocomposite titanate increases with the increase in quantum number S of rare earth ion R^3+^. The microwave dielectric permittivity of LiNiPO_4_ ceramic for LTCC applications is explored in [27]. The relaxation time was demonstrated to vary by TiO_2_ addition. The centimeter-wavelength dielectric permittivity of flower-like NiO structures was studied in [28]. These structures reveal enhanced microwave absorption properties. A new high-performance cobalt-free BaCe_0.16_Y_0.04_Fe_0.8_O_3−δ_ nanocomposite was synthesized and studied [29].

As presented above, a short review of the dielectric properties of rare earth manganates and orthophosphates shows that these properties are valuable in order to understand magnetoelectric behavior. Most of the studies concern polycrystalline or ceramic samples, while nanocomposite samples are less examined. The structure and microwave dielectric properties of nanocomposite manganates with R^3+^: Er, Tb, Yb, and La; titanates R_2_Ti_2_O_7_ with R^3+^: Gd, Tb, Yb, Er, and Sm; and orthophosphates LiCoPO_4_ and LiNi_0.5_Co_0.5_PO_4_ at frequencies of 26 to 38 GHz are studied in this paper. All nanocomposites have been obtained by impregnation of particles in artificial opal matrices. Greatest attention is paid to which type of the frequency dependence of dielectric permittivity is realized, monotonic or nonmonotonic.

## 2. Preparation and Characterization of Nanocomposite Samples

**Figure 1:**
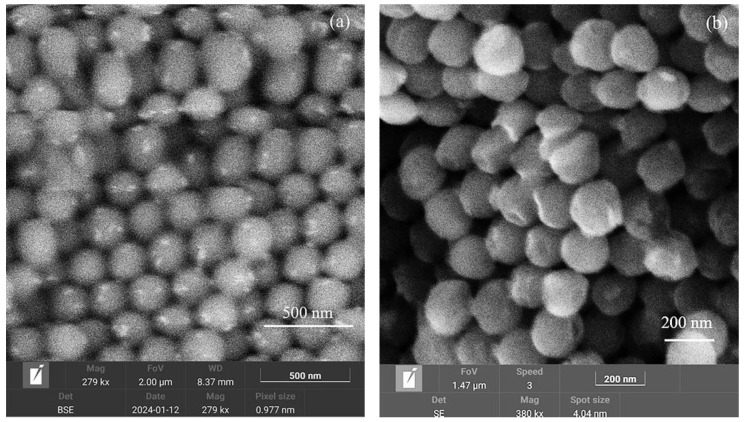
Structure of the nanocomposite with TbMn_2_O_5_ particles (**a**) and of the nanocomposite with LiCoPO_4_ particles (**b**) obtained with the scanning electron microscope Tescan MIRA LMS.

Artificial opal matrices with a submicron SiO_2_ sphere diameter close to 260 nm were chosen as a matrix for nanocomposite samples. The opal matrices were prepared in JSC “Central Research Institute of Technology” Technomash. The nanocomposites were obtained by the impregnation method with subsequent thermal treatment [30]. The matrix was saturated by a water precursor solution. Drying and thermal treatment were performed later. In order to obtain the rare earth manganates, annealing at 900 °C was need. To increase the filling of the inter-spherical voids, the impregnation and thermal treatment procedures were repeated 4–6 times. The structure of the samples was investigated with the scanning electron microscope Tescan MIRA LMS (Tescan, Brno, Czech Republic). An image of the structure of the sample with TbMn_2_O_5_ is shown in Figure 1a. The particles of the embedded substance (they are light in Figure 1a) had either irregular or close to spherical shape. The size of most particles did not exceed 60 nm.

**Figure 2:**
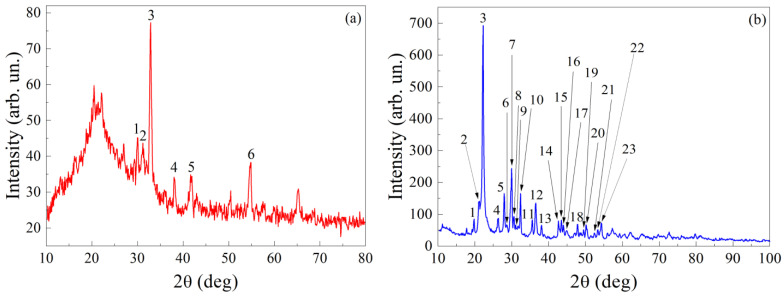
The X-ray diffraction images for samples with TbMn_2_O_5_ (**a**) and LiCoPO_4_ (**b**) particles.

The elemental composition was performed with an EDAX instrument. For the sample with TbMn_2_O_5_ particles, this analysis determined the following elements: Tb, Mn, O, and Si. The elemental composition of this sample is presented in Table 1. An X-ray diffraction image for the sample with TbMn_2_O_5_ particles is shown in Figure 2a. XRD refinement was conducted to identify the detailed structural information and the phase content [31]. The X-ray data for the sample are given in Table 2. The X-ray diffraction data obtained with DRON–3M and XRD-6000 with Cu*K*_α_ irradiation show that, besides SiO_2_ (hexagonal syngony, space group P3_2_21), the following crystalline phases are present in the composite: TbMn_2_O_5_ (orthorhombic syngony, Pbam, 50-0294), in accordance with the ICDD PDF-2 database, and also small amount of Mn_2_O_3_ (cubic syngony, Ia-3, 89-4836).

The crystalline structure of LiMPO_4_ orthophosphates belongs to the olivine type of structure, and it is described by the crystallographic space group *Pnma* [32]. The element cell of orthophosphates contains four Co^++^ or/and Ni^++^ ions. The crystalline structure has a rhombic symmetry. In order to obtain composites, a sample of an opal matrix is placed in Li_3_PO_4_ and Ni_3_(PO_4_)_2_ or Co_3_(PO_4_)_2_ solution, where it is kept for certain number of minutes so that the inter-spherical voids are filled with the solution. After drying, annealing at 900 °C is performed. This procedure is repeated 4–6 times for more infilling of voids by orthophosphate. The method for preparing composite multiferroics in the opal matrices is described in [33].

The structure of the nanocomposite samples with orthophosphate particles has been studied with the scanning electron microscope Tescan MIRA LMS. The results for the sample with LiCoPO_4_ particles are shown in Figure 1b. The embedded substance is found in the inter-spherical voids. Therefore, the maximal size of the particles is less than 60 nm. The shape of the particles is either irregular or close to spherical. The elemental composition obtained with EDAX is presented in Table 3. X-ray structure analysis is carried out in Cu*K*_α_ irradiation. The X-ray diffraction image for samples with LiCoPO_4_ particles is shown in Figure 2b. The X-ray data for the sample with LiCoPO_4_ nanoparticles are presented in Table 4. Silicon dioxide (SiO_2_) is mostly in X-ray amorphous state and partially in the crystalline one. The crystalline phase of SiO_2_ corresponds to cristobalite (tetragonal syngony, P4_1_2_1_2) and tridymite (hexagonal syngony, *P6_3_/mmc*). The introduced substance LiCoPO_4_ or LiNi_0.5_Co_0.5_PO_4_ is related to orthorhombic syngony, *Pnma*. Estimations give sizes of 37–50 nm for the regions of coherent X-ray scattering.

A characterization of the samples of rare earth titanates is presented in [34]. A list of the samples used in this paper is presented in Table 5. The chemical composition of particles in nanocomposites is shown, as well as the number of impregnations applied during preparation. The values of the real *ε*′ and imaginary *ε*″ parts of the dielectric permittivity of the samples averaged over the frequency range 26 to 38 GHz are presented in Table 5.

As a whole, all samples under study have the elemental and phase composition close to the nominal one. The nanoparticles of the embedded substance are located in the inter-spherical voids of an opal matrix. The concentration of the embedded substance is below the percolation threshold. Particles are either irregular in shape or close to spherical.

## 3. Measurement of Microwave Dielectric Permittivity

**Figure 3:**
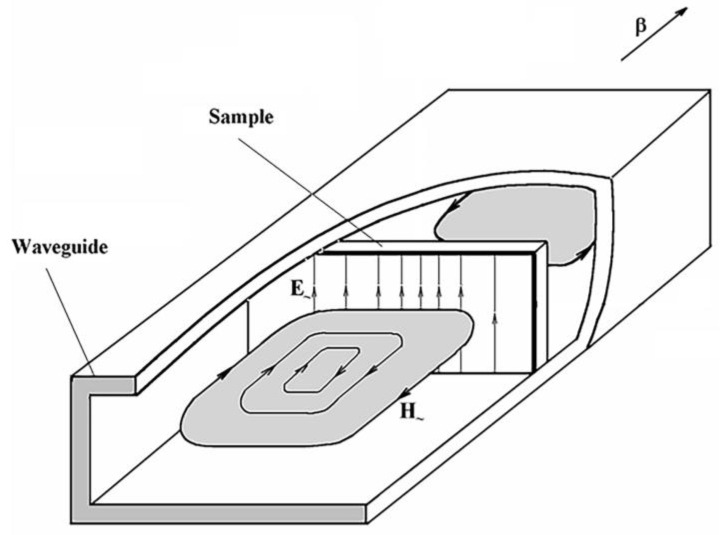
Scheme of microwave fields during the measurements of transmission and reflection coefficients.

The microwave measurements are carried out using the wave transmission/reflection method at room temperature [35]. The measurements are performed in the frequency range 26 to 38 GHz. The scheme of the sample disposition in a waveguide is shown in Figure 3. The power transmission *T* and reflection *R* coefficient modules and their frequency dependences are measured.

The view of the frequency dependence of transmission and reflection coefficients is determined by the following factors: (1) the relation between the sample thickness *d* and the wavelength λ, (2) the value of the absorption coefficient, and (3) the dispersion of TE_10_ mode in the rectangular waveguide. The frequency dependences of transmission and reflection coefficients are used in order to restore the effective dielectric permittivity $\varepsilon =\varepsilon^{\prime }-i\varepsilon^{\prime \prime }$ of a nanocomposite medium [35]. Let us denote the impedance for the part of the waveguide with the sample as $Z_{2}$ and the impedance outside the sample as $Z_{1}$. The complex propagation constant in the sample is indicated as $\beta_{2}=$ $\beta_{2}^{\prime }+i\beta_{2}^{\prime \prime }$. The thickness of the sample equals *d*, and the greater side of the waveguide is *a*. The complex transmission *T* and reflection *R* coefficients can be calculated via the formulas [36,37]

(1) $$T=\frac{1}{\cos\beta_{2}d+\frac{i}{2}\left(\xi +\xi^{-1}\right)\sin\beta_{2}d}$$

(2) $$R=\frac{\frac{i}{2}\left(\xi -\xi^{-1}\right)\sin\beta_{2}d}{\cos\beta_{2}d+\frac{i}{2}\left(\xi +\xi^{-1}\right)\sin\beta_{2}d}$$

where $\xi =Z_{2}/Z_{1}$ is the ratio of the impedances. The propagation constant is calculated as $\beta_{2}=\sqrt{{\left(\frac{\omega }{c}\right)}^{2}\varepsilon \mu -{\left(\frac{\pi }{a}\right)}^{2}}$, where *ε* and *μ* are the effective dielectric permittivity and magnetic permeability of the composite media. We believe that *μ* ≈ ~1 at our frequencies ~30 GHz. Let us designate the experimentally measured transmission coefficient module as $\left|T_{*}\right|$ and the difference between the theoretical transmission coefficient from (1) and the experimental transmission coefficient at a given frequency ω=2πf as $\Delta_{T}=\Delta_{T}\left(\omega ,\varepsilon \right)=\left|T\right|-\left|T_{*}\right|$. In the same way, we can introduce $\Delta_{R}=\Delta_{R}\left(\omega ,\varepsilon \right)=\left|R\right|-\left|R_{*}\right|$ for the reflection coefficient. The value of the complex permittivity $\varepsilon =\varepsilon^{\prime }-i\varepsilon^{\prime \prime }$ is an unknown quantity. Using the least square method, we search for the minimal value of the total approximation error

(3) $${\left(\Delta \left(\varepsilon_{*}^{\prime },\varepsilon_{*}^{\prime \prime }\right)\right)}^{2}=\underset{\varepsilon^{\prime }=\varepsilon_{*}^{\prime }}{\min}\underset{\varepsilon^{\prime \prime }=\varepsilon_{*}^{\prime \prime }}{\min}\;\left[{\left(\Delta_{R}\left(\omega ,\varepsilon^{\prime },\varepsilon^{\prime \prime }\right)\right)}^{2}+{\left(\Delta_{T}\left(\omega ,\varepsilon^{\prime },\varepsilon^{\prime \prime }\right)\right)}^{2}\right]$$

The value $\varepsilon_{*}=\varepsilon_{*}^{\prime }-i\varepsilon_{*}^{\prime \prime }$ obtained as a result is considered an estimate of the dielectric permittivity. The minimization of the error (3) can be performed both in the entire frequency range, in which measurements were performed, and in narrower intervals within it, which can be called windows. By moving such a window over the frequency range, it is possible to determine the frequency dependences of the complex permittivity, rather than its single value, as when estimating over the entire range. Thus, the use of a frequency window makes it possible to identify the dispersion of permittivity of the material under study. The minimal width of the window is selected in such a way that it contains at least one period of oscillation of the frequency dependences of the reflection and transmission coefficients, which can appear due to the partial mismatch of the waveguide measuring line to the load at its output. Averaging the data within the frequency window makes it possible to reduce the influence of these oscillations on the result of the permittivity estimation.

The part of the power dissipated inside the sample is designated as dissipation *D*. The absorbed and scattered power contributes to the dissipation and the power of non-propagating modes transformed from the fundamental waveguide mode TE_10_. The dissipation can be calculated from the transmission and reflection coefficients as

(4) $$D=1-{\left|T\right|}^{2}-{\left|R\right|}^{2}$$

## 4. Dielectric Permittivity of Manganate Composites

**Figure 4:**
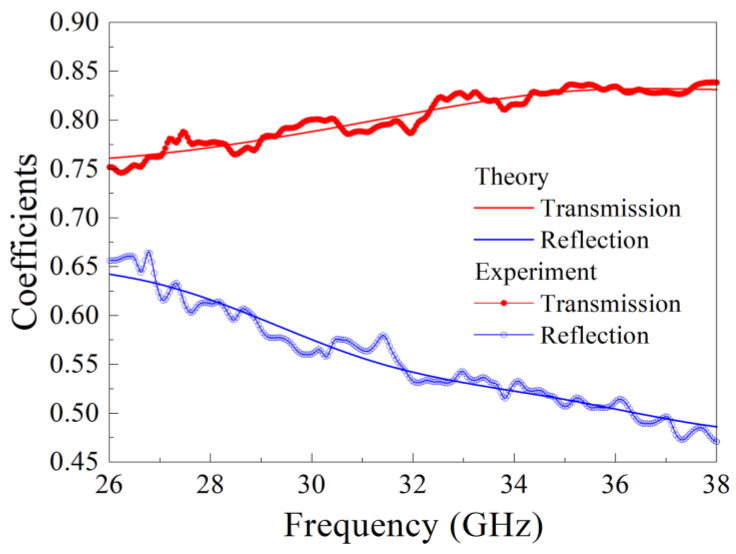
Frequency dependences of transmission and reflection coefficients of electromagnetic waves for a nanocomposite sample with ErMn_2_O_5_ particles: experimental dependences are bold lines, and calculated dependences are thin solid lines.

**Figure 5:**
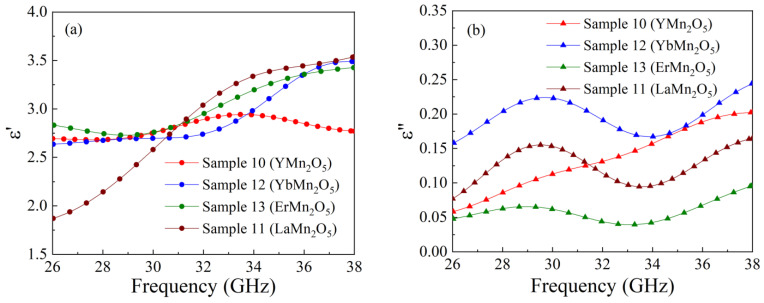
Frequency dependences of real (**a**) and imaginary (**b**) parts of the dielectric permittivity of the composite with manganate particles.

Let us consider the results of the frequency dependences’ measurement of transmission and reflection coefficients for manganate composite samples. These dependences for a nanocomposite sample with ErMn_2_O_5_ particles are shown in Figure 4. It is obvious that the calculated dependence is close to the measured one, and small differences are conditioned by a slight mismatch of the microwave tract. The application of the method described in the above section allows us to restore the frequency dependence of the real and imaginary parts of the complex dielectric permittivity. The results for samples with YMn_2_O_5_, ErMn_2_O_5_, YbMn_2_O_5_, and LaMn_2_O_5_ particles are shown in Figure 5. The designation of the manganate samples is given in Table 5. Within the range concerned, the real part of permittivity *ε*′ of sample 10 with YMn_2_O_5_ particles varies nonmonotonically with the increase in frequency, and it has a maximum close to 33 GHz. The dependences for other samples increase in the frequency range 26 to 38 GHz, but they evidently have maxima at higher frequencies because *ε*′ must tend toward 1 if the frequency rises infinitely. The imaginary part *ε* for samples 11, 12, and 13 is a nonmonotonic function; moreover, for these samples, it has a maximum at frequencies of 30–32 GHz. In the high-frequency region of the range, namely, at frequencies of more than 35 GHz, the *ε*″ function increases again. The inequality *ε*″ << *ε*′ is valid for all samples in the whole frequency range 26–38 GHz. For comparison, the dielectric permittivity of an empty opal matrix is about 2.4–2.5. In principle, a low value of *ε*″ is not surprising for multiferroics; see [38], for example.

## 5. Dielectric Permittivity of Rare Earth Titanate Composites

**Figure 6:**
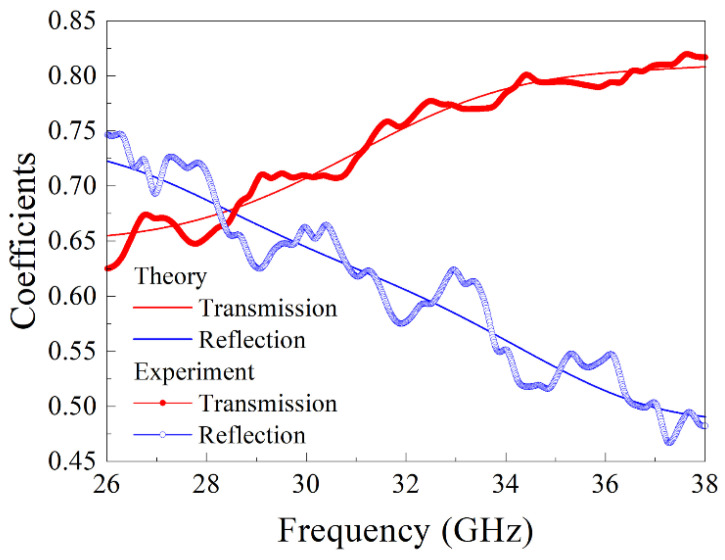
Frequency dependences of transmission and reflection coefficients of electromagnetic waves for a nanocomposite sample with Gd_2_Ti_2_O_7_ particles: experimental dependences are bold lines, and calculated dependences are thin solid lines.

**Figure 7:**
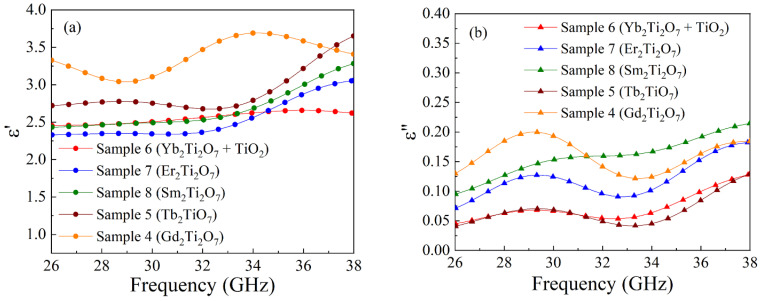
Frequency dependences of real (**a**) and imaginary (**b**) parts of the dielectric permittivity of the composite with rare earth titanates.

The frequency dependences of transmission and reflection coefficients for sample 4 with Gd_2_Ti_2_O_7_ particles are shown in Figure 6. The bold lines indicate the measured dependences, whereas the thin lines are related to the calculated ones via Formulas (1) and (2). The frequency dependences of the real and imaginary parts of the dielectric permittivity of the nanocomposites with rare earth titanate particles are shown in Figure 7, and the averaged ones over the frequency range values are placed in Table 5. The frequency dependences of $\varepsilon^{\prime }$ for samples 4–6 are nonmonotonic within the discussed range. For samples 5, 7, and 8 at frequencies above 35 GHz, an increase in $\varepsilon^{\prime }$ is seen if the frequency rises. Probably, the maximum takes place at higher frequencies. For the imaginary part of the dielectric permittivity, the maximum is found close to 29–30 GHz for samples 4–7. Therefore, the nonmonotonic frequency dependence for both the real and imaginary parts of the dielectric permittivity is observed for almost all samples of rare earth titanates under study.

## 6. Dielectric Permittivity of Orthophosphate Composites

**Figure 8:**
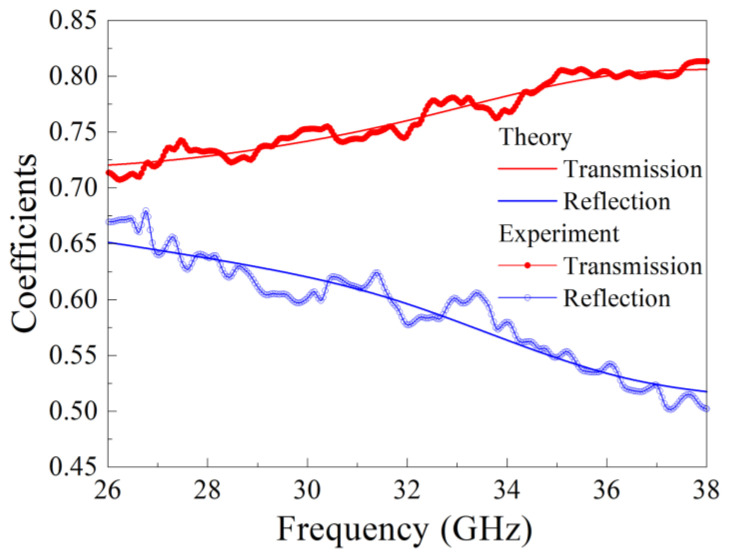
Frequency dependences of transmission and reflection coefficients of electromagnetic waves for nanocomposite sample 1 with LiCoPO_4_ particles: experimental dependences are bold lines, and calculated dependences are thin solid lines.

Let us now discuss the results of measurements of transmission and reflection coefficients for the samples with orthophosphate particles. The measurements are carried out with three samples: with LiCoPO_4_ particles and different impregnations and with the sample of a composite with LiNi_0.5_Co_0.5_PO_4_ particles. Frequency dependences of transmission and reflection coefficients of electromagnetic waves for nanocomposite sample 1 with LiCoPO_4_ particles and 6 impregnations are shown in Figure 8.

**Figure 9:**
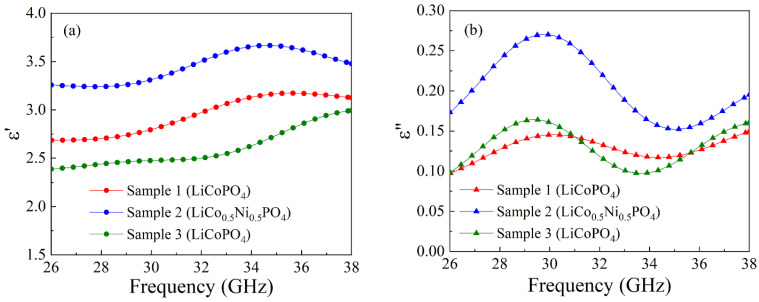
Frequency dependences of real (**a**) and imaginary (**b**) parts of the dielectric permittivity of the composite with orthophosphate particles.

Small differences between the calculated and measured values of the coefficients are caused by the minor mismatch of the tract. Frequency dependences of the real and imaginary parts of dielectric permittivity are shown in Figure 9. The *ε*′ dependence is a nonmonotonic function for samples 1 and 2, and *ε*″ has a maximum for all samples. The inequality *ε*″ << *ε*′ is realized for all samples in the whole frequency range 26–38 GHz. The mean values of the real and imaginary parts of dielectric permittivity averaged over the range 26–38 GHz are presented in Table 5.

## 7. Discussion

From the electromagnetic wave theory in condensed matter is known that the dielectric permittivity possesses dispersion, and its frequency dependence is expressed in terms of the frequencies of the transverse and longitudinal phonons, as follows [1]:

(5) $$\varepsilon (\omega )=\varepsilon^{\infty }+\frac{\varepsilon^{0}-\varepsilon^{\infty }}{1-\omega^{2}/{\omega_{TO}}^{2}}=\varepsilon^{\infty }\frac{\omega_{LO}^{2}-\omega^{2}}{\omega_{TO}^{2}-\omega^{2}}$$

where $\varepsilon^{0}$ and $\varepsilon^{\infty }$ are the isothermal and adiabatic dielectric permittivity, respectively; $\omega_{LO}$ is the frequency of longitudinal phonons; $\varepsilon \left(\omega_{LO}\right)=0$, $\omega_{TO}$ is the frequency of transversal phonons; and $\varepsilon \left(\omega_{TO}\right)=0$. We deal with the frequencies $\omega <<\omega_{LO},\omega_{TO}$. That is why we will analyze the diverse simple models and try to approximate the dependences shown in Figure 5, Figure 7 and Figure 9. The simplest and generally used is the Debye model [39], as follows:

(6) $$\varepsilon \left(\omega \right)=\varepsilon^{\infty }+\frac{\varepsilon^{0}-\varepsilon^{\infty }}{1+i\omega \tau }$$

where τ is the relaxation time. It is adopted in this model that relaxation is described by one oscillator. In the Cole–Cole model, the following expression is proposed [40]:

(7) $$\varepsilon \left(\omega \right)=\varepsilon^{\infty }+\frac{\varepsilon^{0}-\varepsilon^{\infty }}{1+{\left(i\omega \tau \right)}^{1-\alpha }}$$

where α is a coefficient, $\alpha \in \left[0,\;1\right)$. The Davidson–Cole model is close the this one [41]; it looks like

(8) $$\varepsilon \left(\omega \right)=\varepsilon^{\infty }+\frac{\varepsilon^{0}-\varepsilon^{\infty }}{{\left(1+i\omega \tau \right)}^{\beta }}$$

where β is a constant, $\beta \in \left(0,\;1\right]$. The Havriliak–Negami model [42] contains two parameters, *α* and *β*, as follows:

(9) $$\varepsilon \left(\omega \right)=\varepsilon^{\infty }+\frac{\varepsilon^{0}-\varepsilon^{\infty }}{{\left(1+{\left(i\omega \tau \right)}^{1-\alpha }\right)}^{\beta }}$$

where $\alpha \in \left[0,\;1\right)$, $\beta \in \left(0,\;1\right]$. The real and imaginary parts of dielectric permittivity corresponding to (9) can be written in the explicit form (see [43]), as follows:

(10) $$\varepsilon^{\prime }\left(\omega \right)=\varepsilon^{\infty }+\left(\varepsilon^{0}-\varepsilon^{\infty }\right)r^{-\frac{\beta }{2}}\;\cos\left(\beta \theta \right)$$

(11) $$\varepsilon^{\prime \prime }\left(\omega \right)=\left(\varepsilon^{0}-\varepsilon^{\infty }\right)r^{-\frac{\beta }{2}}\;\sin\left(\beta \theta \right)$$

where

(12) $$r={\left[1+{\left(\omega \tau \right)}^{1-\alpha }\sin\left(\frac{\alpha \pi }{2}\right)\right]}^{2}+{\left[{\left(\omega \tau \right)}^{1-\alpha }\cos\left(\frac{\alpha \pi }{2}\right)\right]}^{2}$$

and

(13) $$\theta =\mathrm{atan}\left[\frac{{\left(\omega \tau \right)}^{1-\alpha }\cos\left(\frac{\alpha \pi }{2}\right)}{1+{\left(\omega \tau \right)}^{1-\alpha }\sin\left(\frac{\alpha \pi }{2}\right)}\right]$$

Models (6)–(9) can be generalized for the case of several discrete relaxation times. The sum of several terms is necessary to write corresponding to every relaxation time. For example, Formula (6) for K relaxation times takes the form

(14) $$\varepsilon \left(\omega \right)=\varepsilon^{\infty }+\sum_{k=1}^{K}\frac{\Delta \varepsilon_{k}}{1+i\omega \tau_{k}}$$

where $\Delta \varepsilon_{k}=\varepsilon_{k}^{0}-\varepsilon_{k}^{\infty }$, $\varepsilon^{\infty }=\varepsilon_{K}^{\infty }$, and $\varepsilon^{0}=\varepsilon_{1}^{0}$. Where $\varepsilon^{0}=\varepsilon \left(\omega =0\right)$ and $\varepsilon^{\infty }=\underset{\omega \to \infty }{\lim}\varepsilon \left(\omega \right)$. Let us notice that the following expressions result from (14):

(15) $$\varepsilon^{\prime }\left(\omega \right)=\varepsilon^{\infty }+\sum_{k=1}^{K}\frac{\Delta \varepsilon_{k}}{1+{\left(\omega \tau_{k}\right)}^{2}}$$

(16) $$\varepsilon^{\prime \prime }\left(\omega \right)=\sum_{k=1}^{K}\frac{\omega \tau_{k}\Delta \varepsilon_{k}}{1+{\left(\omega \tau_{k}\right)}^{2}}$$

It is possible to generalize the Formulas (15) and (16) for the case when the continuous function of the relaxation times distribution $\xi \left(\tau \right)$ is discussed against of the discrete relaxation times. Therefore, the integral relations are following from (15) and (16)

(17) $$\varepsilon^{\prime }\left(\omega \right)=\varepsilon^{\infty }+\int_{0}^{\infty }\frac{\xi \left(\tau \right)d\tau }{1+{\left(\omega \tau \right)}^{2}}$$

(18) $$\varepsilon^{\prime \prime }\left(\omega \right)=\int_{0}^{\infty }\frac{\omega \tau \xi \left(\tau \right)d\tau }{1+{\left(\omega \tau \right)}^{2}}$$

which correspond to the Fröhlich model [44]. In the case of the discrete set of relaxation times, when $\xi \left(\tau \right)=\sum_{k=1}^{K}\Delta \varepsilon_{k}\delta \left(\tau -\tau_{k}\right)$, where $\delta \left(x\right)$ is the Dirac delta function, Formulas (17) and (18) go into relations (15) and (16).

One more possibility is to explain the frequency dispersion of permittivity if a stretched exponential decay is realized in the composites under study [45]. The function Φ(t) is introduced in order to describe the time dependence of the electric polarization if the electric field is switched on instantaneously, as follows:

(19) P(t)=P(t→∞)Φ(t)

The dielectric permittivity can be calculated from the time derivative of Φ(t), as follows:

(20) $$\varepsilon^{\prime }\left(\omega \right)-1=(\varepsilon^{\prime }(\omega \to 0)-1)\int_{0}^{\infty }\left(-\frac{d\Phi }{dt}\right)\cos\omega t\;dt$$

(21) $$\varepsilon^{\prime \prime }\left(\omega \right)=(\varepsilon^{\prime }(\omega \to 0)-1)\int_{0}^{\infty }\left(-\frac{d\Phi }{dt}\right)\sin\omega t\;dt$$

The function Φ(t) in the Debye model has the following form:

$$\Phi (t)=\exp\left(-\frac{t}{\tau }\right)$$

and our calculations show that an exponential decay of this sort does not satisfy our experimental data. In the case of stretched exponential decay, Φ(t) is chosen in the form of the Kohlrausch–Williams–Watts function, as follows:

(22) $$\Phi (t)=\exp{\left(-\frac{t}{\tau }\right)}^{\gamma }$$

where γ>0. Under the choice of (22), the real and imaginary parts of dielectric permittivity can be calculated via the Formulas (20) and (21) [19]. In the case of stretched exponential decay, the $\varepsilon^{\prime }$ function decreases more slowly than in the Debye model, and it is possible to diminish $\varepsilon^{\prime \prime }$ to some extent within a definite frequency range.

All examined models (6)–(9), (20), and (21) result in a conclusion that the frequency dependences of the real part of dielectric permittivity are represented by the monotonically decreasing functions. In the case of two or more relaxation times, this dependence can look like a stepwise function also decreasing monotonically; see Formulas (14)–(16). For the composite materials, dielectric permittivity should be considered an effective permittivity. This effective permittivity can be calculated by mixing formulas from the permittivities of components [46]. It is possible to make sure that the Maxwell Garnett and Bruggeman formulas for the effective permittivity also led to a monotonic frequency dependence of $\varepsilon^{\prime }$. Therefore, within the discussed models, we face an irreconcilable contradiction with the experimental fact that the frequency dependence of a real part of dielectric permittivity is a nonmonotonic function for a number of nanocomposite samples of rare earth manganates and titanates, as well as lithium orthophosphates.

**Figure 10:**
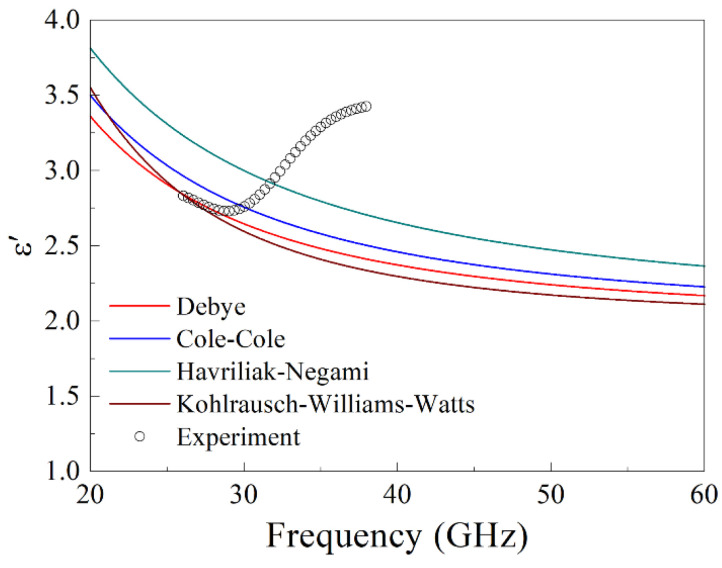
Approximations of the frequency dependence of *ε*′ for sample 12 with YbMn_2_O_5_ particles, which are made using different relaxation models.

As an example, we now consider the results of the approximations of the frequency dependence of the real part of permittivity for sample 12 using the Debye, Cole–Cole, Havriliak–Negami, and Kohlrausch–Williams–Watts models, which are shown in Figure 10.

The experimental dependence being considered here was derived from the results of microwave measurements of transmission and reflection coefficients; see Figure 5. The calculations were performed using Formulas (6), (7), (9) and (20)–(22) for the following parameter values: $\varepsilon^{0}$ = 11.75, $\varepsilon^{\infty }$ = 1, τ = 2.2 · 10^−11^ s, α = 0.02, β = 0.95, γ = 1.3. The choice of this set provides an approximation of the “regular” part of the experimental curve where the permeability decreases with increasing frequency.

Let us discuss why the imaginary part of the dielectric permittivity $\varepsilon^{\prime \prime }$ is essentially less than the real one, i.e., $\varepsilon^{\prime \prime }$ << $\varepsilon^{\prime }$. Of course, we deal now with the frequency range of 26 to 38 GHz. Following the data from Table 5, $\varepsilon^{\prime \prime }$ is only 2–4% from $\varepsilon^{\prime }$ for the samples with ErMn_2_O_5_, Tb_2_Ti_2_O_7_, Yb_2_Ti_2_O_7_ + TiO_2_, and Er_2_TiO_5_ particles.

A possibility to explain the very low values of $\varepsilon^{\prime \prime }$ lies in the analytic properties of the $\varepsilon^{\prime \prime }(\omega )$ function. For the media with spatial dispersion, this function is not a single-valued function of the complex frequency $\dot{\omega }=\omega^{\prime }-i\omega^{\prime \prime }$, and the singularities of a function can present in the upper half plane. Then the Kramers–Kronig dispersion relations in their typical view fail, and the relation between $\varepsilon^{\prime }(\omega )$ and $\varepsilon^{\prime \prime }(\omega )$ can vary. For example, an essential difference between the Debye model and the Kramers-Kronig dispersion relations as regards the decrease in $\varepsilon^{\prime \prime }$ has been observed in optics near the exciton state [47]. Further investigations are required in order to make the type of relaxation clear. Under our conditions, the inequality $q^{\prime }l<<1$ is valid and the spatial dispersion is considered to be weak [48]. Here, $q^{\prime }$ is the real part of the wavenumber, and l is a length characteristic for the space scale of the nanocomposite structure. This is either the size of a separate particle ~60 nm or the period of the matrix structure ~260 nm. These spatial scales are too small in order to destroy the inequality. As known, the long-range crystalline ordering within the regions ≤ 1 mm in length is present in opal artificial crystals [49]. In principle, this extensive spatial scale could be a reason for the spatial dispersion taken into account. Therefore, the relation between the real and imaginary parts of dielectric permittivity can vary.

## 8. Conclusions

The frequency dependences of the transmission and reflection coefficients have been measured, and the complex dielectric permittivity for several nanocomposite rare earth manganates and titanates and Co-Ni lithium orthophosphates has been defined. These objects are chosen for study because they are multiferroics and possess a magnetoelectric effect. The composites are obtained by the impregnation of particles of multiferroics into artificial opal matrices. The dispersion of complex dielectric permittivity is studied at frequencies of 26 to 38 GHz. It has been established that the imaginary part of dielectric permittivity is much less than the real part in this frequency range. A comparison of the frequency dependences of the real and imaginary parts of permittivity with those prescribed by the Debye, Cole–Cole, Havriliak–Negami, and Kohlrausch–Williams–Watts models is performed, and it is demonstrated that all these models fail to satisfactorily describe the dispersion of the dielectric permittivity. In particular, these models cannot explain a nonmonotonic frequency dispersion of the real part of the dielectric permittivity observed for a number of nanocomposite samples of rare earth manganates and titanates and lithium orthophosphates.

## Figures and Tables

**Table 1:** The elemental composition of sample with TbMn_2_O_5_ particles.

Element	Type of Line	Wt.%	At.%
O	K	51.46	66.97
Si	K	42.76	31.70
Mn	L	2.31	0.88
Tb	M	3.47	0.45

**Table 2:** The X-ray data for sample with TbMn_2_O_5_ nanoparticles.

Peak’s Number	Experimental Data					Data from ICDD PDF-2					
	Intensity, arb. un.	Intensity, %	Bragg Angle 2θ, deg.	Peak Semiwidth, deg.	Interplane Space, Å	Mn_2_O_3_—Cubic Syngony, Space Group Ia-3 (89-4836)			TbMn_2_O_5_—Orthorhombic Syngony, Space Group Pbam (50-0294)		
						d, Å	hkl	I, %	d, Å	hkl	I, %
1	17.93	23	29.820	0.396	2.9934				3.0400	121	65
2	17.30	22	30.967	0.396	2.8851				2.8580	211	100
3	77.25	100	32.688	0.396	2.7370	2.7152	222	100	2.7430	220	11
4	6.28	8	37.827	0.253	2.3761	2.3515	400	13.7	2.3740	131	9
5	10.48	14	41.745	0.417	2.1617				2.1540	311	30
6	15.51	20	54.716	0.341	1.6760	1.6627	440	39.9	1.6740	420	23
7	12.00	16	65.155	0.289	1.4304	1.4180	622	17.6	1.4140	060	18

**Table 3:** The elemental composition of a sample with LiCoPO_4_ particles.

Element	Type of Line	Wt.%	At.%
O	K	56.33	69.83
Si	K	41.28	29.15
P	K	0.69	0.45
Co	K	1.70	0.57

**Table 4:** The X-ray data for the sample with LiCoPO_4_ nanoparticles.

Peak’s Number	Experimental Data					Data from ICDD PDF-2								
	Intensity, arb. un.	Intensity, %	Bragg Angle 2θ, deg.	Peak Semiwidth, deg.	Interplane Space, Å	SiO_2_ (Cristobalite)— Tetragonal Syngony, Space Group P4_1_2_1_2 (89-3434)			LiCoPO_4_— Orthorhombic Syngony, Space Group Pnma (85-0002)			SiO_2_ (Quartz)— Hexagonal Syngony, Space Group P3_2_21 (86-1565)		
						d, Å	hkl	I, %	d, Å	hkl	I, %	d, Å	hkl	I, %
1	34.99	5	19.523	0.328	4.5427									
2	105.34	15	20.908	0.328	4.2448				4.2610	101	67.5			
3	692.02	100	21.984	0.328	4.0394	4.0281	101	100				4.0722	100	22.1
4	35.17	5	26.172	0.271	3.4017				3.4583	111	81.1			
5	81.98	12	27.823	0.220	3.2035							3.2191	011	100
6	14.53	2	28.528	0.220	3.1259									
7	105.78	15	29.732	0.157	3.0020				2.9821	211	10.3			
8	25.48	4	30.343	0.157	2.9430				2.9599	020	64.0			
9	10.94	2	30.847	0.157	2.8960									
10	60.06	9	32.143	0.157	2.7821	2.8340	102	11.4	2.7527	301	24.5			
11	35.20	5	35.237	0.316	2.5446				2.5600	220	2.4			
12	97.48	14	36.186	0.316	2.4800	2.4793	200	13.5	2.4960	311	100			
13	27.59	4	37.776	0.316	2.3792				2.3419	002	17.5	2.3511	110	12.0
14	40.96	6	42.391	0.225	2.1303	2.1114	211	2.6				2.1461	111	5.3
15	39.12	6	43.172	0.225	2.0935				2.1305	112	16.7			
16	22.70	3	43.733	0.225	2.0680									
17	20.00	3	44.618	0.225	2.0290	2.0140	202	1.9	2.0157	321	4.4	2.0361	200	7.8
18	35.55	5	47.539	0.374	1.9109	1.9246	113	4.8	1.9319	420	2.2	1.8986	021	1.5
19	18.82	3	49.189	0.374	1.8506	1.8660	212	4.8						
20	38.70	6	49.957	0.374	1.8239				1.8380	230	12.1			
21	12.33	2	52.210	0.243	1.7504							1.7522	112	8.6
22	22.29	3	53.130	0.243	1.7222				1.7291					
23	50.37	7	54.088	0.243	1.6940	1.6871	203	2.3						

**Table 5:** Designation and mean values of dielectric permittivity of composites.

No. of Sample	Chemical Composition of Samples	Number of Impregnations	<*ε*′>	<*ε*″>
1	LiCoPO_4_	6	2.94	0.13
2	LiCo_0.5_Ni_0.5_PO_4_	5	3.45	0.21
3	LiCoPO_4_	5	2.59	0.13
4	Gd_2_Ti_2_O_7_	5	3.17	0.16
5	Tb_2_Ti_2_O_7_	5	2.73	0.07
6	Yb_2_Ti_2_O_7_ + TiO_2_	5	2.52	0.07
7	Er_2_Ti_2_O_7_	5	2.39	0.12
8	Sm_2_Ti_2_O_7_	5	2.52	0.16
9	Er_2_TiO_5_	5	2.74	0.023
10	YMn_2_O_5_	10	2.36	0.13
11	LaMn_2_O_5_	5	2.81	0.12
12	YbMn_2_O_5_	6	2.85	0.2
13	ErMn_2_O_5_	6	2.72	0.06

## Data Availability

The original contributions presented in this study are included in the article. Further inquiries can be directed to the corresponding author.
